# Immunization with a Bacterial Lipoprotein Establishes an Immuno-Protective Response with Upregulation of Effector CD4+ T Cells and Neutrophils Against Methicillin-Resistant *Staphylococcus aureus* Infection

**DOI:** 10.3390/pathogens9020138

**Published:** 2020-02-20

**Authors:** Zhenzi Peng, Duo-Yao Cao, Hui-Ya Wu, Suguru Saito

**Affiliations:** 1 Institute of Medical Sciences, Xiangya Hospital, Central South University, Changsha 410008, China; zzpivy@csu.edu.cn; 2 Department of Bio Medical Science, Cedars-Sinai Medical Center, Los Angeles, CA 90048, USA; 3 College of Animal Science and Technology, Northwest A&F University, Shaanxi 712100, China; 9527caojunn@gmail.com; 4 College of Health Science, Trans World University, Yunlin 640, Taiwan; Wuhuiya0815@gmail.com; 5 Biofluid Biomarker Center, Graduate School of Medical and Dental Sciences, Niigata University, Niigata 9518510, Japan

**Keywords:** *S. aureus*, lipoprotein, dendritic cell, Th1, Th17, neutrophil, MRSA, acute skin inflammation, immunization

## Abstract

*Staphylococcus aureus* (*S. aureus*) is a commensal bacterium in the human body; however, the bacterium frequently generates serious inflammation and infectious diseases. Some strains of *S. aureus*, such as methicillin-resistant *Staphylococcus aureus* (MRSA), are still a serious problem in public health facilities. Thus, an effective protection strategy is eagerly expected for the prevention and cure of MRSA infection. Here, we report that a specific fraction of an *S. aureus* lipoprotein (SA-LP) established a protective response against MRSA infection. The fractionated *S. aureus* lipoprotein SA-LP-F2, which is contained in 30–50 kDa of crude *S. aureus* lipoprotein (SA-LP-C), effectively activated dendritic cells (DCs) and the SA-LP-F2-pulsed DCs generated IFN-γ+CD4+ T (Th1) and IL-17A+CD4+ T (Th17) cells by in vitro antigen presentation. The SA-LP-F2 immunization upregulated the Th1 and Th17 populations so that MRSA colonization on the skin was suppressed during the challenge phase with MRSA. By following the effector T cell upregulation, the neutrophil function, which was a substantial effector cell against MRSA, was also enhanced in the SA-LP-F2-immunized mice. Finally, we found that the protective effect of SA-LP-F2 immunization was maintained for at least 90 days because the immunized mice continued to show a protective response during the MRSA challenge period. In the MRSA challenge, reactivated Th1 and Th17 populations were maintained in the SA-LP-F2-immunized mice as compared to naive mice. In addition, the neutrophil population was also upregulated in the mice. The memory CD4+ T cell (central memory T; T_CM_ and effector memory T; T_EM_) population was established by SA-LP-F2 immunization and was maintained at higher levels than usual. Taken together, our findings may provide a breakthrough in the establishment of an immunization strategy against MRSA infection.

## 1. Introduction

*Staphylococcus aureus* (*S. aureus*) is a Gram-positive commensal bacterium in the human body [1]. *S. aureus* colonization is regularly controlled at a minimum level and is benign in terms of host immunity [2]. However, once growth is promoted by several factors, the bacterium has a critical influence on the host [3]. For instance, immunodeficiency and synergistic infections with other pathogens that disturb a healthy immune environment or serious traumatic injuries are typical situations that cause dramatic colonization of *S. aureus* [4]. Once the bacterium starts abnormal colonization, it is very hard to regulate the growth, especially for patients with a critical background such as underlying disease [5]. Antibiotics are frequently used to deal with abnormal *S. aureus* colonization; however, the treatment itself is generally limited to prevent the emergence of resistant bacteria [6]. Hence, the strategy against *S. aureus* infection should be expected to shift to prevention rather than treatment. 

A specific characteristic of a commensal bacterium is the ability to maintain a suitable balance between innate and adaptive immunity in order to survive. Immunization against *S. aureus* or other commensal bacteria is a difficult task in immunology. Many studies were performed to establish effective immunization against *S. aureus* infection using the bacterial components [7,8,9]. These studies identified the effective substances in *S. aureus* to activate innate immune cells, such as dendritic cells (DCs) and macrophages; however, these responses did not lead to a subsequent direct immunological protection against *S. aureus* because they failed to establish an effective adaptive immune response. Recently, methicillin-resistant *Staphylococcus aureus* (MRSA) infection has become a critical problem not only in clinical settings but also in daily life [10]. To prevent serious situations related to MRSA infection, the establishment of an optimized immunization protocol and strategy is eagerly expected.

Here, we showed that an immunization with *S. aureus* lipoproteins establishes effector T cells based on an adaptive immune response accompanied by the upregulation of neutrophil function against MRSA infection. The specific fraction of *S. aureus* lipoprotein (SA-LP-F2), which is contained in 30–50 kDa of crude *S. aureus* lipoprotein (SA-LP-C) [11], showed a stronger activation compared with other fractions in murine primary DCs. The SA-LP-F2 intraperitoneal (IP) injection promoted IFN-γ+CD4+ T (Th1) and IL-17A+CD4+ T (Th17) cell upregulation in an antigen-specific manner in both the spleen and lymph node (LN) as compared to the naive environment. From this evidence, we performed immunization with SA-LP-F2 with the aim of establishing an effective anti-bacterial immunity. The SA-LP-F2-immunized mice established an effective immune response against MRSA as compared to SA-LP-C and heat-killed *S. aureus* (HK-SA) immunization in the acute skin inflammation model. The skin lesion area of the MRSA-injected site was significantly reduced in SA-LP-F2-immunized mice as compared to naive or other immunized mice. The bacterial colony forming unit (CFU) on the lesion skin was also significantly reduced in the immunized mice. The immune response was mainly based on Th1 and Th17 upregulation at a glance; however, neutrophils were suspected as another key player in this response because the population of skin-accumulated neutrophil was also significantly expanded by MRSA challenge in SA-LP-F2-immunized mice. In vivo neutrophil depletion completely abolished the anti-bacterial response in SA-LP-F2-immunized mice even though Th1 and Th17 populations were maintained in the immunized mice. Finally, we found that the SA-LP-F2 immunization effect was maintained for at least 90 days in the anti-bacterial immunity. The immunized mice effectively eliminated the challenged MRSA with reactivation of Th1 and Th17 as well as upregulation of neutrophil accumulation in the skin on days 30, 60, and 90 post SA-LP-F2 immunization. The SA-LP-F2 immunization established memory CD4+ T cells (central memory T; T_CM_ and effector memory T; T_EM_), which eventually became effector CD4+ T cells by reactivation during MRSA infection. 

Thus, immunization with the specific fraction of the *S. aureus* lipoprotein enhanced both innate and adaptive immunity against MRSA infection, but it is still unclear which substance in the fraction has the most effective immunogenicity. However, our result provides a clue to the investigation of the *S. aureus-*originated substance which can be used for anti-bacterial immunization such as targeting MRSA infection.

## 2. Materials and Methods

### 2.1. Mice

C57BL/6J-specific pathogen-free (SPF) mice were purchased from the Jackson Laboratory (Bar Harbor, ME, USA). Gender-matched mice between 8 and 12 weeks of age were used for each experiment. Immunization was performed with SA-LP (10 ug), SA-LP-F2 (10 ug) or HK-SA (10^7^ CFU/mL) in 50% of complete Freund's adjuvant (CFA) by injection into the hind food pad (final volume; 100 ul). Incomplete Freund’s adjuvant (IFA) or aluminum (Alm) was also used in some immunizations. Some mice received phosphate-buffered saline (PBS) injection as a vehicle control, and these mice were used as naive mice. An acute skin inflammation model was established by intradermal (ID) injection of live-MRSA (10^7^ CFU/mL). The skin lesion area and the bacterial colony forming unit (CFU) were calculated by following the method described in a previous report [12]. All the experimental materials and protocols were approved by the animal care and use committee of Central South University (Protocol No.; 201503302) and Northwest A&F University (Protocol No.; 15-10-874).

### 2.2. Reagents and Antibodies

Pam3CSK4, phorbol 12-myristate 13-acetate (PMA) and ionomycin were purchased from Sigma Aldrich (St. Louis, MO, USA). Dispase, collagenase, DNase, CellROX^TM^Green, FluoReporter™ fluorescein isothiocyanate (FITC), a Protein Labeling Kit, Ovalbmin (OVA), and FITC-conjugated OVA were purchased from Thermo Fisher Scientific (Waltham, MA, USA). Percoll was purchased from GE Healthcare (Chicago, IL, USA). A Cytofix/Cytoperm kit with GolgiStop^TM^ was purchased from BD Bioscience (Franklin Lakes, NJ, USA). Recombinant murine granulocyte macrophage-colony stimulating factor (rmGM-CSF) and recombinant murine interleukin-2 (rmIL-2) were purchased from Peprotech (Rocky Hill, NJ, USA). Anti-CD11c (N418), anti-CD11b (M1/70), anti-CD80 (16-10A1), anti-CD86 (GL-1), anti-MHC-II (I-A/I-E; M5/114.15.2), anti-mouse Ly-6G/Ly-6C (Gr-1) (RB6-8C5), anti-CD45 (30-F11), anti-CD3 (17A2), anti-CD4 (GK1.5), anti-Interferon gamma (IFN-γ) (XMG1.2), anti-IL-17A (BL168), and anti-CD16/CD32 (2.4G2) (93) were purchased from Biolegend (San Diego, CA, USA). Anti-CD4 (GK1.5), anti-IFN-γ (H22), anti-IL4 (BVD6-24G2), anti-IL-17A (17F3) mAb, and anti-Ly-6G (1A8) were purchased from Bio X Cell (West Lebanon, NH, USA). The isotype-matched control for each antibody was purchased from the same company.

### 2.3. S. aureus Culture 

Frozen *S. aureus* (MRSA; USA300) stock was purchased from the American Type Culture Collection (ATCC) (Manassas, VA, USA). The bacteria were thawed on ice, and then transferred to a tryptic soy broth (TSB; BD bioscience, Franklin Lakes, NJ, USA) and cultured at 37 °C for 18 h with shaking. The CFUs were calculated in each culture. Heat-killed *S. aureus* (HK-SA) was prepared by heating at 95 °C for 30 min. The heated *S. aureus* suspension was centrifuged at 10,000 g for 1 min to harvest the bacterial cells, and then the cell pellet was resuspended in PBS.

### 2.4. Lipoprotein Preparation 

Lipoprotein was isolated from *S. aureus* (MRSA; USA300) by following a method described in previous reports with some modifications [11,13]. Briefly, cultured *S. aureus* (10^7-8^ CFU/mL) were harvested by centrifuging at 5000 g for 20 min. The pellet was washed twice with 20 mM Tris-HCl (pH 8.0). The pellet was resuspended in 20 mM Tris-HCl (pH 8.0), and then the bacterial cells were crushed with 0.3 mm stainless beads. The treated suspension was centrifuged at 5000 g for 20 min, and then the supernatant was harvested as the protein suspension. The suspension was mixed with 100% ethanol and kept at −20 °C overnight. The sample was centrifuged at 12,000 g for 15 min, and then the precipitated pellet was washed with 80% ethanol and centrifuged again at 12,000 g for 5 min. The precipitated pellet was dissolved in 1 M urea/50 mM Tris-HCl and 50 mM ethylenediamine tetra acetic acid (EDTA) (pH 8.0) (crude protein extract; CPE) [11,14]. Triton X-114 was added to the protein suspension (final 1%), and then the suspension was incubated at 4 °C with gentle mixing overnight. The incubated suspension was heated at 37 °C to allow formation of the micelle phase containing lipoproteins. The micelle phase was extracted and the lipoprotein (crude *S. aureus* lipoprotein; SA-LP-C) was harvested by following a method for CPE precipitation. The SA-LP-C was separated into four fractions (SA-PL-F1: greater than 50 kDa, SA-LP-F2: 30–50 KDa, SA-LP-F3: 10–30 kDa, SA-LP-4: less than 10 kDa) using a molecular weight cut-off filter (Amicon Ultra, Zeba™ Spin Desalting Columns; Thermo Fisher Scientific) by following a method described in a previous publication [11]. 

### 2.5. Mouse Primary Cell Isolation and Blood Leukocyte Preparation

Lymph node (LN) cells and splenocytes were prepared from inguinal LNs and spleen, respectively, by following a method described in a previous report [11]. Briefly, the extracted LNs and spleen were crushed on a 70 μm cell strainer with cell culture medium (RPMI 1640 supplemented with 10% fetal bovine serum (FBS), 100 U/mL penicillin, and 100 mg/mL streptomycin). The cell suspension was filtered through a 70 μm cell strainer again and then washed with cell culture medium. The cell suspension was treated with 1xRBC lysis buffer at room temperature (RT) for 10 min. After being washed twice with the cell culture medium, the cells were used as LN cells and splenocytes, respectively. Skin leukocytes were isolated by following a method described in a previous report with slight modification [11]. Briefly, the extracted skin piece was washed with tissue washing buffer (RPMI 1640 supplemented with 10% fetal bovine serum (FBS), 10 mM 4-(2-hydroxyethyl)-1-piperazineethanesulfonic acid (HEPES), 100 U/mL penicillin, 100 mg/mL streptomycin) at 37 °C for 30 min with gentle shaking. The skin piece was incubated at 37 °C for 30 min with dispase working solution (tissue washing buffer containing 0.25 mg/mL of dispase) to separate the epidermal and dermal sheets. These sheets were chopped with scissors, and then the skin fragments were incubated at 37 °C for 30 min in collagenase working solution (tissue washing buffer containing 1 mg/mL collagenase and 0.01% DNase). The digested skin pieces were filtered through a 70 μm cell strainer, and then the flow through was passed through a 5 mL syringe with a 22 G needle to form a single cell suspension. Mouse bone marrow leukocytes were obtained from the tibia and femur by following a method described in a previous report [11]. Briefly, the cells were flushed out from the tibia and femur by a 10 ml syringe with a 27 G needle containing cell culture medium. The cell suspension was filtered through a 70 μm cell strainer and washed once with cell culture medium, and then the cells were treated with the 1xRBC lysis buffer at RT for 10 min. After being washed twice with cell culture medium, the cells were used as bone marrow leukocytes. 

Splenic DCs were isolated from splenocytes by using a MagniSort^TM^ Mouse CD11c Positive Selection Kit (Thermo Fisher Scientific, Waltham, MA, USA). Naive CD4+ T cells were isolated from splenocytes by using a MagniSort^TM^ Mouse CD4 naive T cell Enrichment Kit (Thermo Fisher Scientific, Waltham, MA, USA). CD4+ T cells were isolated from splenocytes by using a MagniSort^TM^ Mouse CD4 T cell Enrichment Kit (Thermo Fisher Scientific, Waltham, MA, USA). Neutrophils were isolated from bone marrow by using an EasySep™ Mouse Neutrophil Enrichment Kit (STEMCELL TECHNOLOGIES, Vancouver, BC, Canada). The whole procedure for the cell isolation kit was performed by following the manual. The cell purity was checked by flow cytometry, and the purified cell suspension with greater than 90% purity was used for the experiment. Blood leukocytes were prepared from 100 μl of peripheral blood. The blood sample was diluted with 1xRBC lysis buffer and was incubated at RT for 10 min. After being washed twice with PBS/2% FBS, the precipitated cells were used as peripheral blood leukocytes.

### 2.6. Mouse Bone Marrow Dendritic Cell (BMDC) Preparation

Mouse BMDCs were prepared by following a method described in a previous report [11]. On day 0, 2.0 × 10^6^ of bone marrow leukocytes were suspended in 10 mL of DC culture medium (cell culture medium containing 20 ng/mL of rmGM-CSF), and the cells were seeded on a 100 mm dish. On day 3, 10 mL of the fresh DC culture medium was added to the cultured cells. On days 6 and 8, half of the cultured medium was collected and centrifuged, and then the cell pellets were resuspended in 10 mL of the fresh DC culture medium. The cell suspension was returned to the original plate. On day 10, cells were ready to use for each experiment. The percentage of CD11c+ cells was checked by flow cytometry, and the culture with more than 90% of CD11c+ cells was used for the experiment. 

### 2.7. DC Stimulation Assay

Naive BMDCs and splenic DCs were stimulated with SA-LP (1 μg/mL), fractionated SA-LP (SA-LP-F1 to 4; 1 μg/mL), HK-SA (10^6^ CFU/mL), and Pam3CSK4 (500 ng/mL) in cell culture medium at 37 °C overnight. The cultured medium was harvested for cytokine detection by Enzyme-Linked Immunosorbent Assay (ELISA) and stored at −80 °C until use. 

### 2.8. In Vivo Antigen Uptake Assay

FITC-labeled SA-LP-F2 (10 ug), SA-LP-C (10 ug) or OVA (10 ug) was ID injected into mice back skin. After 48 h, skin-draining LNs (skin-dLNs) were corrected, and the cells were isolated for analysis. The antigens taking up migratory DCs (mDCs) (FITC+ in CD45+CD11b+MHC class II hi population) were detected by flow cytometry.

### 2.9. T Cell Restimulation Assay

The splenocytes were isolated from immunized mice and were restimulated with antigen (SA-LP; 10 ug/mL, SA-LP-F2; 10 ug/mL, and HK-SA; 10^6^ CFU/mL) in cell culture medium at 37 °C for 72 h. The cultured medium was harvested for cytokine measurement by ELISA and stored at −80 °C until use.

### 2.10. Antigen Presentation and T Cell Differentiation Assay

Isolated splenic DCs (2.0 × 10^4^) were mixed with splenic naive CD4+ T cell (1.0 × 10^5^) in cell culture medium supplemented with IL-2 (10 ng/mL). The antigen (SA-LP; 1 ug/mL, SA-LP-F2; 1 ug/mL and HK-SA; 10^6^ CFUs/mL) was added to the culture, and then the cells were incubated at 37 °C for 72 h. Anti-IL4 mAb (10 ng/mL) and anti-IL-17A mAb (10 ng/mL) were added to the culture for Th1 differentiation. Anti-IFN-γ mAb (10 ng/mL) and anti-IL-4 mAb (10 ng/mL) were added to the culture for Th17 differentiation. In the last 6 h of the coculture, the proliferated cells were restimulated with PMA (100 ng/mL) and ionomycin (1 μg/mL), and protein transportation was inhibited with Golgi Stop^TM^ (BD Bioscience) in the last 2 h. The proliferated T cells were characterized by flow cytometry.

### 2.11. Bacteria Killing Assay

MRSA (10^6^ CFU/mL) was applied to the cell culture medium, and then neutrophils (1.0 × 10^6^, isolated from naive or SA-LP-F2-immunized mice) were seeded into the culture. The culture was incubated at 37 °C for 6 h. After the incubation, the MRSA CFUs were measured, and reactive oxygen species (ROS) production in neutrophil was analyzed by flow cytometry. 

### 2.12. Flow Cytometry

Cell surface markers and intracellular cytokines were analyzed by flow cytometers (FACSCant and LSR-II; BD Biosciences, Franklin Lakes, NJ, USA) with the fluorochrome-conjugated monoclonal antibodies described in reagents and antibodies. The cells were initially incubated with FcR blocker (anti-CD16/32; 2.4G2) at 4 °C for 10 min. For surface marker staining, the cells were incubated with antibody at 4 °C for 30 min. Intracellular cytokine staining was performed by using a Cytofix/CytoPerm Kit by following the manual. Briefly, the cells stained with the antibody for the surface marker were fixed and permeabilized at 4 °C for 20 min. The cells were incubated with the antibody for intracellular cytokine staining at 4 °C for 30 min. For ROS production assay, the neutrophils were stained with CellROX^TM^Green in the presence of antibodies for the extracellular marker at 37 °C for 30 min. The target population was detected by following a gating strategy indicated in Figure S1. All data were analyzed by BD FACS Diva (BD bioscience, Franklin Lakes, NJ, USA) or FlowJo (Tree Star; Ashland, OR, USA).

### 2.13. Cytokine Measurement by Enzyme-Linked Immunosorbent Assay (ELISA)

The cytokines (TNF-α, IFN-γ, and IL-17A) produced from the stimulated cell were measured by the ELISA Ready-SET-Go!™ Kit (Thermo Fisher Scientific, Waltham, MA, USA) for each target. The whole procedure was performed by following the manual.

### 2.14. Statistical Analyses 

Two-way analysis of variance (ANOVA) was used to analyze the data for significant differences. Values of *p < 0.05, **p < 0.01, and ***p < 0.001 were regarded as significant.

## 3. Results

### 3.1. SA-LP-F2 Activates DCs, and the Ability Is Stronger Than That of Other Fractions 

In a previous report, we found that the crude *S. aureus* lipoprotein (SA-LP-C) induced an immune response in a murine acute skin inflammation model [11]. The effector T cells were generated by activated DCs with the SA-LP-C as the antigen in mice and in an in vitro study. The SA-LP-C can be separated into several fractions depending on the size (SA-LP-F1: greater than 50 kDa, SA-LP-F2: 30–50 kDa, SA-LP-F3: 10–30 kDa, SA-LP-F4: less than 10 kDa) (Figure S2), so we further characterized the immunogenicity of each fraction, especially for primary DCs, to understand the physiological function of the SA-LPs.

**Figure 1:**
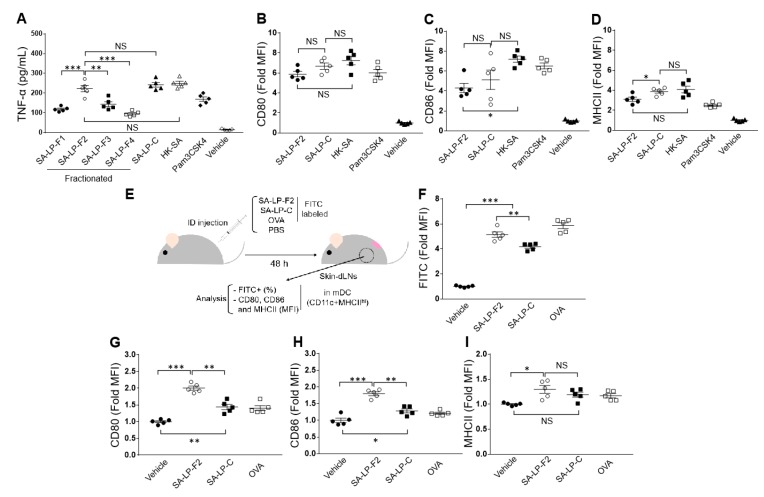
SA-LP-F2 activated primary DCs, and the ability was stronger than that observed for other fractions. (**A**) Cytokine production in stimulated DCs. Murine splenic DCs (1.0 × 10^6^) were stimulated with fractionated SA-LP (SA-LP-F1 to 4; 1 ug/mL), SA-LP-C (1 ug/mL), HK-SA (10^6^ CFU/mL), and pam3CSK4 (500 ng/mL) at 37 °C overnight. The cultured medium was harvested, and then TNF-α production was measured by ELISA. (**B–D**) Profiling of surface activation marker in stimulated DCs. Murine splenic DCs (1.0 × 10^6^) were stimulated with SA-LP-F2 (1 ug/mL), SA-LP-C (1 ug/mL), HK-SA (10^6^), and pam3CSK4 (500 ng/mL) at 37 °C overnight. The surface expressions of CD80 (**B**), CD86 (**C**), and MHC class II (**D**) were analyzed by flow cytometry. (**E**) Design of in vivo antigen uptake assay. FITC-labeled SA-LP-F2 (10 ug), SA-LP-C (10 ug), and OVA (10 ug) were ID injected into mice back skin. After 48 h, skin-dLNs were harvested, and mDCs were analyzed by flow cytometry. **F**) FITC (antigen), (**G**) CD80, (**H**) CD86, and (**I**) MHC class II MFI in mDCs. PBS was used for vehicle control in the experiments (A–I). Data are shown as the mean ± SEM of five samples. Two-way analysis of variance (ANOVA) was used to analyze data for significant differences. Values of *p < 0.05, **p < 0.01, and ***p < 0.001 were regarded as significant. NS; Not significant.

Murine splenic DCs were stimulated with the fractionated SA-LP (SA-LP-F1 to 4) as well as SA-LP-C. HK-SA and Pam3CSK4, which is a bacterial lipopeptide, were used as positive control. SA-LP-F1, 3, and 4 induced almost similar level of TNF-α production in the DCs (Figure 1A). On the other hand, SA-LP-F2-stimulated DCs produced significantly higher amounts of TNF-α as compared to the cells stimulated with other fractionated SA-LPs (Figure 1A). The cytokine production was almost the same as SA-LP-C stimulation in the DCs (Figure 1A). BMDCs were also stimulated with these SA-LPs. In this assay, the difference in TNF-α production between SA-LP-F2 and other SA-LPs was not dramatic; SA-LP-F2 produced a significantly larger amount of TNF-α as compared to SA-LP-F1 and 4 (Figure S3). In addition, the SA-LP-F2-mediated cytokine production was significantly lower than that of SA-LP-C stimulation (Figure S3). This result is consistent with our previous report [11], but the trend was different from that of splenic DCs (Figure 1A).

The cell surface activation markers, such as CD80, CD86, and MHC class II, were markedly upregulated in the SA-LP-F2 stimulated DCs as compared to naive cells (Figure 1B–D). However, the levels were very similar those of SA-LP-C or HK-SA stimulated DCs.

To investigate the physiological response of DCs against SA-LPs, we performed in vivo antigen uptake assay (Figure 1E). The ID-injected FITC-labeled SA-LPs were captured by DCs, and the DCs migrated into skin-dLNs as migratory DCs (mDCs). The FITC MFI in mDC was significantly increased in the mice that received SA-LP-F2 injection as compared to SA-LP-C-injected mice (Figure 1F). Both CD80 and CD86 expressions were significantly upregulated in the SA-LP-F2 captured mDCs as compared to other mDCs (Figure 1G,H). However, MHC class II expression was not different in mDCs between SA-LP-C- and SA-LP-F2-injected mice (Figure 1I).

Taken together, these results show that SA-LP-F2 activated primary DCs and induced cytokine production from the cells. The upregulation of DC activation by SA-LP-F2 was more significant in primary cells and the physiological environment than in in vitro culture.

### 3.2. Fractionated S. aureus Lipoprotein Induces Effector T Cell Differentiation

**Figure 2:**
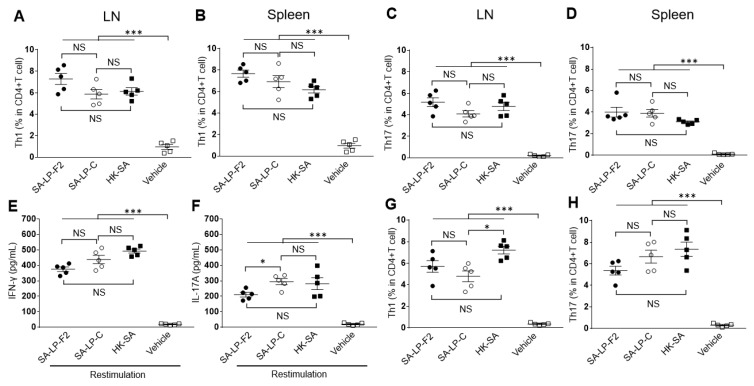
SA-LP-F2 generated effector CD4+ T cells. **(A–D)** The population of effector CD4+ T cells in the SA-LP-F2 IP-injected mice. The mice received IP injection of SA-LP-F2 (10 ug), SA-LP-C (10 ug), and HK-SA (10^7^ CFU/mL) at 50% of CFA. After 7 days, the populations of Th1 (**A,B**) and Th17 (**C,D**) in both the spleen and LNs were analyzed by flow cytometry. (**E–F**) Cytokine production in restimulated splenocytes. The splenocytes (3.0 × 10^6^/mL) isolated from SA-LP-F2 IP-injected mice were restimulated with SA-LP-F2 (10 ug/mL), SA-LP-C (10 ug/mL), and HK-SA (10^6^ CFU/mL) at 37 °C for 72 h. IFN-γ (**E**) and IL-17A (**F**) production in the cultured was measured by ELISA. (**G–H**) In vitro antigen presentation of effector CD4+ T cell generation. The splenic DCs (2.0 × 10^4^) and naïve CD4+ T cells (1.0 × 10^5^) were mixed in the presence of SA-LP-F2 (1 ug/mL), SA-LP-C (1 ug/mL) or HK-SA (10^6^ CFU/mL) at 37 °C for 72 h. The proliferated T cells were characterized for Th1 (**G**) and Th17 (**H**) by flow cytometry. Data are shown as the mean ± SEM of five samples. Two-way ANOVA was used to analyze data for significant differences. Values of *p < 0.05, **p < 0.01, and ***p < 0.001 were regarded as significant. NS; Not significant.

To investigate the antigenicity of SA-LP-F2, we analyzed the effector T cell population in the mice that received IP injection of SA-LP-F2. The IFN-γ+CD4+ T cell (Th1) population was increased in SA-LP-F2-injected mice as compared to naive mice after 7 days of the procedure (Figure 2A,B). In addition, the IL-17A+CD4+ T cell (Th17) population was also established in the SA-LP-F2-injected mice at the same time point (Figure 2C,D). The responses were observed in both the spleen and LNs. The populational upregulation of these effector T cells was at a similar level as that in the mice that received SA-LP-C and HK-SA IP injection.

To confirm the generation of effector T cells in an SA-LP-F2-specific manner, we performed restimulation assay using splenocytes isolated from the SA-LP-F2-injected mice. The splenocytes produced IFN-γ and IL-17A in the restimulation with SA-LP-F2, which meant that the SA-LP-F2 specifically responding T cell populations were established in the mice (Figure 2E,F). Interestingly, the restimulation with SA-LP-C or HK-SA also showed a similar level of IFN-γ and IL-17A production in the culture. In addition, we performed in vitro antigen presentation to naive CD4+ T cells using SA-LP-F2 as an antigen. The SA-LP-F2-pulsed DCs generated both Th1 and Th17 populations in the culture (Figure 2G,H). SA-LP-C and HK-SA-pulsed DCs showed a similar percentage of effector CD4+ T cell generation.

Although SA-LP-C and HK-SA generated effector T cells in the IP-injected mice and the T cells could be restimulated with SA-LP-F2 with specificity, SA-LP-C and HK-SA induced critical inflammatory responses in the mice. The neutrophil population was significantly increased in the spleen and peripheral blood in SA-LP-C- or HK-SA-injected mice as compared to SA-LP-F2-injected mice 24 h post IP (Figure S4A,B). The percentage of neutrophil reached approximately 20–30% in the spleen and approximately 30–50% in peripheral blood in SA-LP-C- and HK-SA-injected mice, respectively; however, the average percentage was approximately 10% in both SA-LP-F2 injected and control mice (Figure S4A,B). In addition, the serum TNF-α level was also significantly increased in the mice that received SA-LP-C and HK-SA injection as compared to SA-LP-F2-injected mice (Figure S4C). These side effects in SA-LP-C- or HK-SA-injected mice were critical even though they were not as severe as those observed in LPS IP-injected mice, which were similar to septic shock.

Taken together, the results indicate that SA-LP-F2 administration induces effector CD4+ T cell differentiation in an antigen-specific manner. In addition, the treatment did not induce critical inflammatory side effects during the establishment of the effector CD4+ T cell population in the mice.

### 3.3. Immunization with SA-LP-F2 Suppresses Acute Skin Inflammation by MRSA Infection

**Figure 3:**
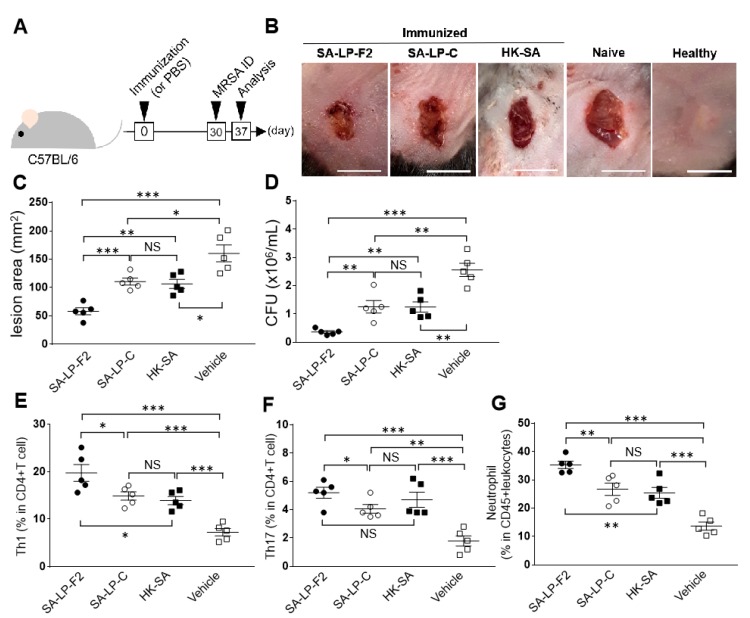
SA-LP-F2 immunization established a protective effect against MRSA infection. (**A**) The experimental design of immunization and MRSA infection. The mice were immunized with SA-LP-F2 (10 ug), SA-LP-C (10 ug) or HK-SA (10^7^ CFU/mL). After 30 days, MRSA (10^7^ CFU/mL) challenge was performed by ID injection into back skin. After 7 days, the mice were sacrificed and used for analysis. (**B**) The skin condition of the MRSA-infected site. Bar = 10 mm. (**C**) The size of the skin lesion area in MRSA-infected mice. (**D**) Bacterial CFUs on the lesion skin. (**E–G**) The population on accumulated immune cells in the lesion skin. The skin piece was corrected from the lesion area, and then the populations of Th1 (**E**), Th17 (**F**), and neutrophils (**G**) were analyzed by flow cytometry. Data are shown as the mean ± SEM of five samples and are representative of at least five independent experiment. Two-way ANOVA was used to analyze data for significant differences. Values of *p < 0.05, **p < 0.01, and ***p < 0.001 were regarded as significant. NS; Not significant.

To evaluate the efficiency of the effector CD4+ T cell generation by SA-LP-F2 immunization, we established a murine acute skin inflammation model by MRSA ID injection into flank skin [11] and investigated the grade of skin inflammation and bacterial colonization on the skin (Figure 3A). For the immunization, the mice received SA-LP-F2 injection into the hind food pad. Then, 21 days after the immunization, Th1 and Th17 populations were increased in the mice (Figure S5A,B). The efficiency of immunization in terms of effector CD4+ T cell generation was compared by using different adjuvants. CFA showed the highest percentage of Th1 and Th17 induction in the SA-LP-F2-immunized mice (Figure S5A,B). 

The mice first received SA-LP-F2, SA-LP-C or HK-SA immunization, and then MRSA was challenged by ID injected into the back skin to induce bacterial colonization and acute skin inflammation (Figure 3A). Seven days after MRSA administration, the skin showed a wide lesion area from MRSA infection in naive mice (Figure 3B). On the other hand, the skin injury was suppressed in SA-LP-C- or HK-SA-immunized mice (Figure 3B). SA-LP-F2-immunized mice showed a much smaller lesion area in the MRSA challenge (Figure 3B). The grade of skin inflammation was quantitatively evaluated by the size of the lesion area. The skin lesion area was significantly reduced in SA-LP-C- or HK-SA-immunized mice as compared to naive mice; however, there was no difference between these two immunization groups (Figure 3C). On the other hand, the SA-LP-F2-immunized mice showed a smaller lesion size than naive mice as well as SA-LP-C- and HK-SA-immunized mice (Figure 3C). Skin-colonized bacterial CFUs were consistent with the grade of skin injury by MRSA challenge. The SA-LP-F2-immunized mice showed the fewest CFUs in the lesion area as compared to other groups of mice (Figure 3D).

The populations of skin that accumulated T cells and neutrophils were also investigated in the MRSA-challenged mice. Similar to the response in the IP injection (Figure 2A,B), Th1 and Th17 populations increased in the immunized mice skin after MRSA challenge; however, there was no obvious difference in the Th17 population between the immunization methods (Figure 3F), while the Th1 population in SA-LP-F2 -mmunized mice clearly increased in MRSA-challenged skin (Figure 3E). Interestingly, the population of skin that accumulated neutrophils was significantly increased in immunized mice as compared to naive mice, and SA-LP-F2-immunized mice showed the largest upregulation of the population (Figure 3G).

Thus, SA-LP-F2 immunization established effector CD4+ T cell upregulation in the mice, and the response protected the host from MRSA infection accompanied with neutrophil accumulation in the inflamed skin. 

### 3.4. CD4+ T Cell Depletion Abolishes the Anti-Bacterial Immunity Established by SA-LP-F2 Immunization 

**Figure 4:**
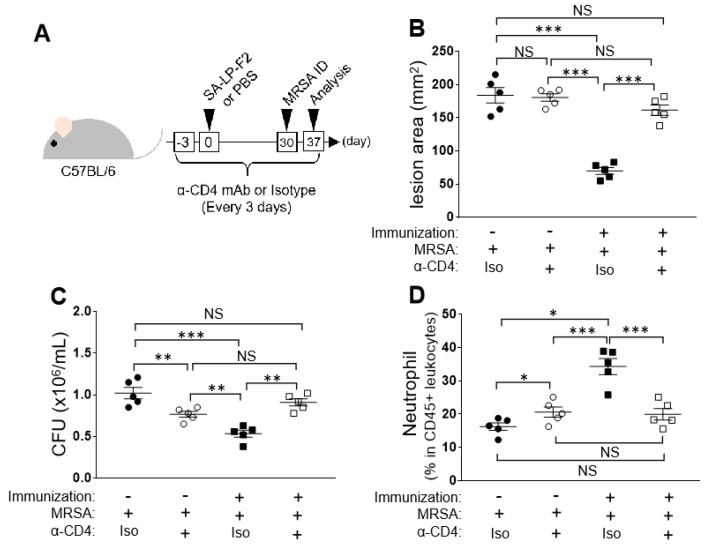
Depletion of CD4+ T cell lacked the immunization effect of SA-LP-F2 in anti-bacterial immunity. (**A**) The experimental design of CD4+ T cell depletion in immunization and MRSA challenge. The mice were immunized with SA-LP-F2 (immunization (+)). Some mice received PBS injection as a naive control (immunization (-)). Thirty days after the immunization, the mice received MRSA challenge in the back skin by ID injection. The mice also received anti-CD4 mAb (or isotype Ab for control) every 3 days during the experimental period. Seven days after MRSA infection, the mice were sacrificed and used for analysis. (**B**) The skin lesion area in MRSA-infected mice. (**C**) Bacterial CFUs on the lesion skin. (**D**) The population of accumulated neutrophils in the lesion skin. The skin piece was corrected from the MRSA-infected site, and then the neutrophil population was analyzed by flow cytometry. Data are shown as the mean ± SEM of five samples. Two-way ANOVA was used to analyze data for significant differences. Values of *p < 0.05, **p < 0.01, and ***p < 0.001 were regarded as significant. NS; Not significant.

We hypothesized that the SA-LP-F2 specific effector CD4+ T cells upregulate anti-bacterial immunity under MRSA challenge in the immunized mice. To investigate the T cell contribution, we depleted CD4+ T cells and performed SA-LP-F2 immunization as well as MRSA challenge in the mice (Figure 4A). Because in vivo depletion of CD4+ T cell by anti-CD4 mAb eliminated almost all CD4+ T cells, we could not detect Th1 and Th17 populations in SA-LP-F2-immunized mice (Figure S6A,B).

The protective effect of SA-LP-F2 immunization was abolished in the CD4+ T cell-depleted mice under the MRSA challenge. Both the skin lesion area and skin-colonized bacterial CFUs were almost identical between CD4+ T cell-depleted SA-LP-F2-immunized mice and naive mice (Figure 4B,C). However, the mice that received administration of isotype Ab still showed a strong anti-bacterial response such that the skin lesion and bacterial CFUs were greatly reduced (Figure 4B,C). The neutrophil accumulation was also suppressed by CD4+ T cell depletion on the MRSA-challenged skin in SA-LP-F2-immunized mice (Figure 4D), while the mice that received isotype Ab injection showed a significantly higher percentage of skin with accumulated neutrophils than CD4+ T cell-depleted mice (Figure 4D).

Thus, the SA-LP-F2 immunization-originated effector CD4+ T cells are indispensable factors to inhibit MRSA colonization. In addition, the T cell population eventually induced neutrophil accumulation in the anti-bacterial response. 

### 3.5. Neutrophil Depletion Abolished the Enhancement of Anti-Bacterial Immunity in SA-LP-F2-Immunized Mice

**Figure 5:**
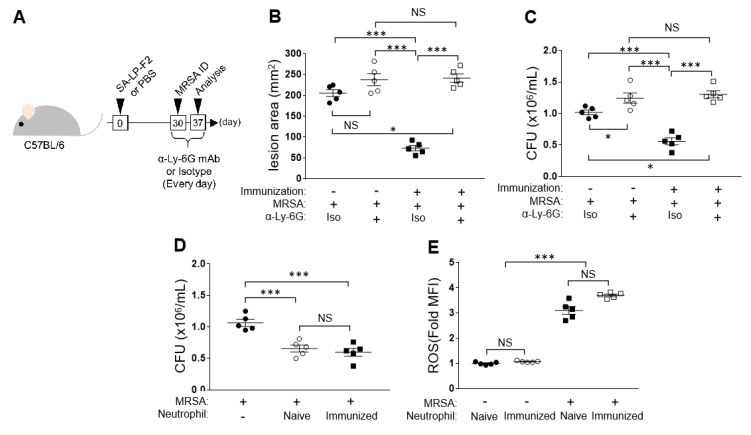
Neutrophils were important players in the enhancement of the anti-bacterial response in SA-LP-F2-immunized mice; however, the original function was not upregulated by the immunization. (**A**) The experimental design of neutrophil depletion in immunization and MRSA challenge. The mice received immunization with SA-LP-F2 (immunization (+)). Some mice received PBS injection as a naive control (immunization (-)). Thirty days after the immunization, the mice received MRSA challenge in the back skin by ID injection. The mice also received anti-Ly-6G mAb (or isotype Ab for control) every day during the experimental period. The neutrophil depletion was confirmed in peripheral blood by flow cytometry (data not shown). Seven days after MRSA infection, the mice were sacrificed and used for analysis. (**B**) The skin lesion area in MRSA-infected mice. (**C**) Bacterial CFUs on the lesion skin. (**D–E**) In vitro bacterial killing and ROS production assay. Neutrophils were isolated from naive or SA-LP-F2-immunized mice BM. The neutrophils (1.0 × 10^6^) were coincubated with MRSA (10^6^ CFU/mL) at 37 °C. The bacteria CFUs were measured at 6 h (**D**), and ROS production in neutrophils was analyzed by flow cytometry at 30 min (**E**). Data are shown as the mean ± SEM of five samples. Two-way ANOVA was used to analyze data for significant differences. Values of *p < 0.05, **p < 0.01, and ***p < 0.001 were regarded as significant. NS; Not significant.

Since we found increased skin-accumulated neutrophils in SA-LP-F2-immunized mice under MRSA challenge (Figure 3G), we investigated the contribution of neutrophils to the anti-bacterial immunity. The neutrophils were depleted by mAb in both naive and SA-LP-F2-immunized mice, and then the skin lesion area and bacterial CFUs in the MRSA-infected site were analyzed (Figure 5A).

Neutrophil depletion aggravated skin inflammation in the MRSA-infected site of naive mice because the lesion area was enlarged in the mice (Figure 5B). In SA-LP-F2-immunized mice, the anti-bacterial response was also abolished by neutrophil depletion, so the skin lesion area was a similar size as that in naive mice (Figure 5B). The suppressive effect of SA-LP-F2 immunization in MRSA colonization was also abolished by neutrophil depletion. The bacterial CFUs increased in neutrophil-depleted mice, even those that received SA-LP-F2 immunization (Figure 5C). 

We next investigated the direct influence of SA-LP-F2 immunization on the neutrophil function against bacteria. Neutrophils were isolated from both naive and SA-LP-F2-immunized mice, and the cells were used for bacteria killing assay. Neutrophils that originated from naive and immunized mice showed almost identical ability in bacterial killing; nearly 40% of MRSA was eliminated in the culture (Figure 5D). In addition, the ROS production showed a similar level of upregulation in the neutrophils that originated from naive and SA-LP-F2-immunized mice under MRSA exposure (Figure 5E).

Taken together, the results show neutrophil is an important player in the SA-LP-F2 immunization-based upregulation of the anti-bacterial response. However, the original ability of neutrophils was not modified by the immunization.

### 3.6. The Long-Term Effect of SA-LP-F2 Immunization in the Anti-Bacterial Response 

**Figure 6:**
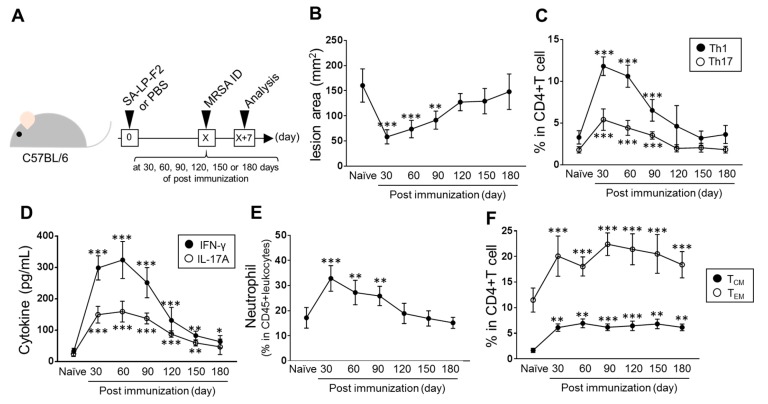
The long-term effect of SA-LP-F2 immunization against MRSA infection. (**A**) The experimental design of immunization and MRSA infection. The mice received immunization with SA-LP-F2 (10 ug). After 30, 60, 90, 120, 150 or 180 days, MRSA (10^6^ CFU/mL) challenge was performed by ID injection into back skin. After 7 days, the mice were sacrificed and used for analysis. (**B–F**) The time-dependent duration of SA-LP-F2 immunization in anti-bacterial immunity. The skin lesion area (**B**) was measured, and spleen Th1 and Th17 (**C**) and skin-accumulated neutrophils (**E**) were analyzed by flow cytometry. The splenocytes were isolated from the MRSA-challenged mice, and then the cells were restimulated with SA-LP-F2 (100 ug/mL) at 37 °C or 72 h. The cytokine production was measured by ELISA (**D**). The population of memory CD4+ T cells in SA-LP-F2-immunized mice after MRSA challenge. Splenic CD4+ T_CM_ and T_EM_ were analyzed by flow cytometry. The PBS-injected mice were used as naïve control in all the experiments. Data are shown as the mean ± SEM of five samples (**A–E**). Two-way ANOVA was used to analyze data for significant differences. Values of *p < 0.05, **p < 0.01, and ***p < 0.001 were regarded as significant. NS; Not significant.

To evaluate the effective duration of SA-LP-F2 immunization in anti-bacterial immunity, we challenged MRSA in the immunized mice at 30, 60, 90, 120, 150, and 180 days post immunization (Figure 6A). The skin lesion area was significantly reduced in SA-LP-F2-immunized mice compared with naive mice in the MRSA challenge at 30, 60, and 90 days post immunization (Figure 6B). However, the lesion size was not significantly reduced in the MRSA-challenged mice at 120, 150, and 180 days post SA-LP-F2 immunization as compared to naive mice (Figure 6B). The T cell population in the MRSA-infected mice was analyzed to evaluate the recalled SA-LP-F2-specific effector CD4+ T cell. The populations of Th1 and Th17 were highly maintained in SA-LP-F2-immunized mice at 30, 60, and 90 days post immunization in the MRSA challenge (Figure 6C).

To assess the effector CD4+ T cell function, splenocytes isolated from MRSA-challenged mice were restimulated with SA-LP-F2. The production of Th1 and Th17 cytokines, such as IFN-γ and IL-17A, was strongly upregulated in the culture prepared from SA-LP-F2-immunized mice as compared to naive mice, and the levels reached a peak in the mice that received MRSA challenge 60 days after SA-LP-F2 immunization (Figure 6D). The population of neutrophil, as a substantial effector cell in anti-bacterial immunity, was also analyzed in the MRSA-challenged mice. The neutrophil accumulation on the MRSA-challenged skin was promoted in the SA-LP-F2-immunized mice (Figure 6E). The response showed significance in the mice challenged with SA-LP-F2 in the immunization period for 30–90 days. The immunological kinetics of neutrophil exactly matched the transition of the size of the skin lesion area in the MRSA-challenged site (Figure 6B) as well as the effector CD4+ T cell response (Figure 6C,D). 

The memory T cell population was also analyzed in the SA-LP-FS-immunized mice after MRSA challenge. We found that both effector memory CD4+ T cell (T_EM_; CD4+CD62L-CD44hi) and central memory CD4+ T cell (T_CM_; CD4+CD62L+CD44hi) populations were significantly increased in the SA-LP-F2-immunized mice as compared to naive mice at every MRSA challenge time point (Figure 6F). The memory T cell upregulation was maintained by SA-LP-F2 immunization even by day 180 post immunization. 

Thus, SA-LP-F2 immunization established anti-bacterial immunity with a long-term effect by maintaining effector CD4+ T cells and neutrophil response.

## 4. Discussion

The *S. aureus* cell wall is composed of various structural components such as peptidoglycan (PGN), lipoteichoic acid (LTA), and lipoproteins [15,16,17]. Several studies aimed to identify the useful substance in these structural components for immunization against bacterial infection; however, almost all studies did not achieve the establishment of an effective strategy in the immunization. In fact, the immunization strategies using bacterial components have several side effects and lack a long-term immunization effect [18,19]. Furthermore, the exact immune response by the immunization is difficult to characterize in individuals [20,21].

In this study, we found the specific effect of the *S. aureus* lipoprotein fraction sized 30–50 kDa, called SA-LP-F2, in the establishment of anti-bacterial immunity. We showed that SA-LP-F2 possessed a strong ability to induce effector CD4+ T cell-based activation of neutrophil function in anti-bacterial immunity (Figure 3, Figure 4, Figure 5 and Figure 6). This is a valuable advantage in this field, because our data suggested that CD4+ T cells and neutrophils were exact target cells to emphasize the function in the elimination of MRSA by SA-LP-F2 immunization. Especially for effector CD4+ T cells, the response was highly specific because the T cell population was restimulated by SA-LP-F2 itself and was recalled by MRSA challenge (Figure 2E,F, Figure 3E,F, and Figure 6C,D). In addition, the immunization formed T_CM_ and T_EM_ pools in the immunized mice, and the populations were probably recalled in MRSA challenge even though it was few month after the initial immunization (Figure 6F).

Although SA-LP-F2 is a mixture of the *S. aureus* lipoproteins, the fraction showed fewer side effects, such as neutrophil increasing in blood and lymphoid organs as well as upregulation of serum inflammatory cytokine levels compared with SA-LP-C- and HK-SA-injected mice (Figure S4A–C). This was an unexpected response because SA-LP-F2 activated splenic DCs as well as primary mDCs (Figure 1B–D,G–I), and a fraction of the induced inflammatory cytokines in splenic DCs (Figure 1A). The most effective lipoprotein or lipopeptide in the SA-LP-F2 fraction to establish the effector CD4+ T cell pool in the immunized mice remains unclear. In fact, several *S. aureus* lipoproteins were identified as a single component able to activate immune cells in an independent manner. These lipoproteins (SAOUHSC_00634, 02650, and 02699), which were identified from fraction 30–35 kDa of crude *S. aureus* lipoprotein, induced TNF-α production in human peripheral blood mononuclear cells (PBMCs) [13,22]. However, these substances failed to induce Th1 and Th17 differentiation in both in vitro antigen presentation and in vivo immunization (unpublished data). We also confirmed that one of the synthetic lipoproteins, which was originally identified in SA-LP-C, markedly activated both BMDCs and splenic DCs. However, the immunization by the lipoprotein failed to upregulate the effector CD4+ T cell population in the immunized mice (unpublished data). Hence, we concluded that the substance that activates immune cells cannot easily be adapted for immunization use. Further studies are required to identify the most effective single component which exactly establishes the T cell-based adaptive immune response in the immunization. Once the component is identified, the strategy will achieve great progress in the immunization for anti-bacterial immunity.

**Figure 7:**
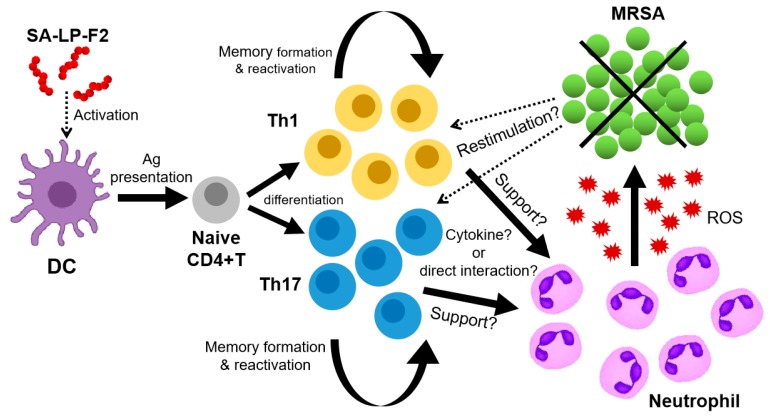
Immunological network in SA-LP-F2-immunized mice under anti-bacterial immunity. SA-LP-F2 is captured by DCs, and then the DCs are activated and prime naive CD4+ T cell by presenting SA-LP-F2 as an antigen. The naive CD4+ T cells are differentiated into both Th1 and Th17. The populations form memory T cell pools in the immunized mice. During the MRSA challenge phase, Th1 and Th17 populations are reactivated. Neutrophils work as substantial effector cells in MRSA elimination; however, the function is upregulated by SA-LP-F2 specifically responding to Th1 and Th17 in the MRSA challenge. Eventually, the orchestrated effector CD4+ T cells and neutrophils show the upregulation of MRSA elimination and the protective effect against the bacterial infection.

When we found the disappearance of the SA-LP-F2 immunization effect in the anti-bacterial response by in vivo CD4+ T cell depletion, we thought that the SA-LP-F2-specific effector CD4+ T cell population was the most important factor in the immunization (Figure 4). This should be true in one of the aspects; however, the major effector was neutrophil in the anti-bacterial response of SA-LP-F2-immunized mice (Figure 3G and Figure 4D). In vivo neutrophil depletion also completely abolished the beneficial effect of SA-LP-F2 immunization in MRSA infection (Figure 5). In addition, neutrophil’s immunological kinetics completely corresponded with those of effector CD4+ T cells in MRSA-infected mice (Figure 6C–E). Furthermore, in vitro bacteria killing assay using neutrophils isolated from both naive and immunized mice showed a similar response in these two different cell sources (Figure 5 D,E). From these results, the SA-LP-F2-specific Th1 and Th17 were indispensable populations, while the functional upregulation of neutrophils by the effector CD4+ T cells was also a critical factor in the anti-bacterial immunity under SA-LP-F2 immunization. The effector CD4+ T cell population might support neutrophil function in the immunized mice for MRSA elimination (Figure 7). A previous report showed that Th1 and Th17 regulated the innate immune system and bacterial clearance [23]. In addition, the possibility of interaction between effector T cells and neutrophils to promote the neutrophil function has been reported [23,24,25]. This evidence supports our findings in SA-LP-F2 immunization. In fact, other T cell subsets, such as IFN-γ+CD8+ T (cytotoxic T lymphocyte; CTL), gamma-delta T (γδT), and natural killer T (NKT) cells, were increased in the lymphoid organs of SA-LP-F2-immunized mice (unpublished data) as well as in the skin of SA-LP-C ID-injected mice [12]. The detailed analysis of these subsets might provide a further interesting response related to neutrophils or other innate immune cells in SA-LP-F2 immunization-based anti-bacterial immunity. 

Taken together, the results indicate that *S. aureus* lipoprotein immunization induces an orchestrated immune response between innate and adaptive systems in the immunized mice. Our approach in the establishment of anti-bacterial immunity has not been completed, and many points must be optimized. However, the possibility of *S. aureus* lipoprotein-based immunization was clearly shown in this study.

## Supplementary Materials

The following are available online at https://www.mdpi.com/2076-0817/9/2/138/s1, Figure S1: Gating strategy in flow cytometry analysis, Figure S2: SA-LP-induced TNF-α production in murine BMDCs, Figure S3: The substance-dependent inflammatory response in the mice, Figure S4: The difference in the adjuvant effect in effector CD4+ T cell generation by SA-LP-F2 immunization, Figure S5: The efficiency of in vivo CD4+ T cell depletion in SA-LP-F2-immunized mice.
