# Supplementary material for: Immunization with a Bacterial Lipoprotein Establishes an Immuno-Protective Response with Upregulation of Effector CD4+ T Cells and Neutrophils Against Methicillin-Resistant Staphylococcus aureus Infection
Source: Pathogens. 2020 Feb 20;9(2):138. doi: 10.3390/pathogens9020138 (PMC7169464; doi:10.3390/pathogens9020138)
Supplement: Supplementary file 1 [file pathogens-09-00138-s001.pdf]

Supplementary materials

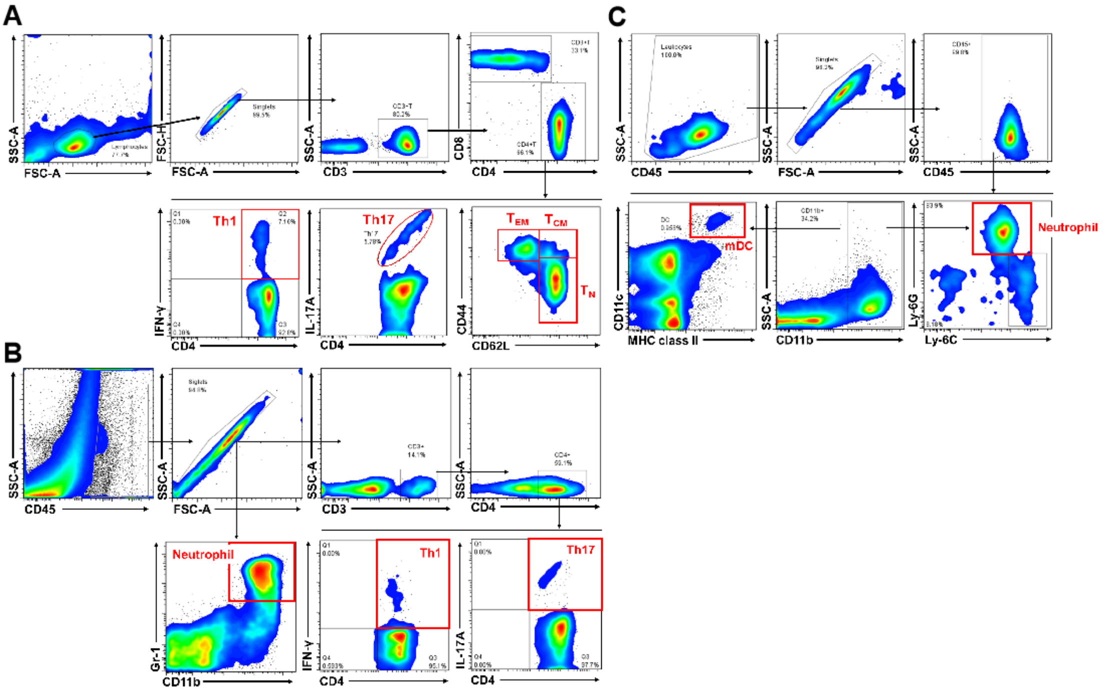

Figure S1. Gating strategy in flow cytometry analysis. A) Analysis for Th1, Th17 and memory CD4+ T cell (in spleen and LNs). B) Analysis for Th1, Th17 and neutrophil (in skin). C) Analysis for neutrophil (in spleen, blood) and mDC (in LNs).

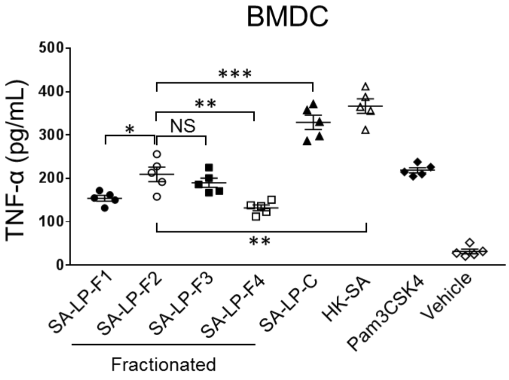

Figure S2. SA-LPs induced TNF-*α* production in murine BMDCs. BMDCs ($1.0x10^6$) were stimulated with fractionated SA-LP (F1-4) (1 ug/mL), SA-LP-C (1 ug/mL), HK-SA (10^6 CFU/mL) and pam3CSK4 (500 ng/mL) at 37°C for overnight. Some samples were tread with PBS as vehicle control. The cultured medium was harvested, then the cytokine concentration in the medium was measured by ELISA. Data are shown as the mean ± SEM of five samples. Two-way analysis of variance (ANOVA) was used to analyze data for significant differences. Values of *p<0.05 , **p<0.01 ***p<0.001 were regarded as significant. NS; Not significant.

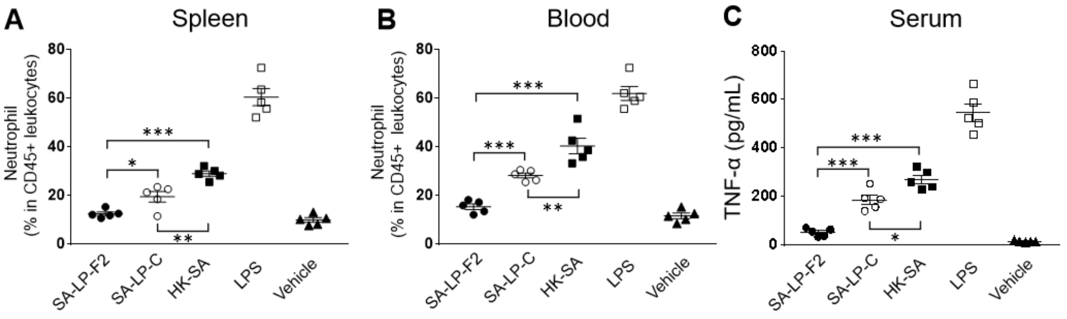

Figure S3. The substance dependent inflammatory response in the mice. A-C) The mice were received IP injection of SA-LP-F2 (10 ug), SA-LP-C (10 ug) or HK-SA (10^6 CFU/mL) respectively. LPS (1mg) was used for a positive control and PBS was used for a vehicle control. After 24 h of the injection, the nutrophil population in spleen (A) and blood (B) were analyzed by flow cytometry. The serum TNF-α was measured by ELISA (C). Data are shown as the mean ± SEM of five samples (A-C). Two-way analysis of variance (ANOVA) was used to analyze data for significant differences. Values of *p<0.05 , **p<0.01, and ***p<0.001 were regarded as significant.

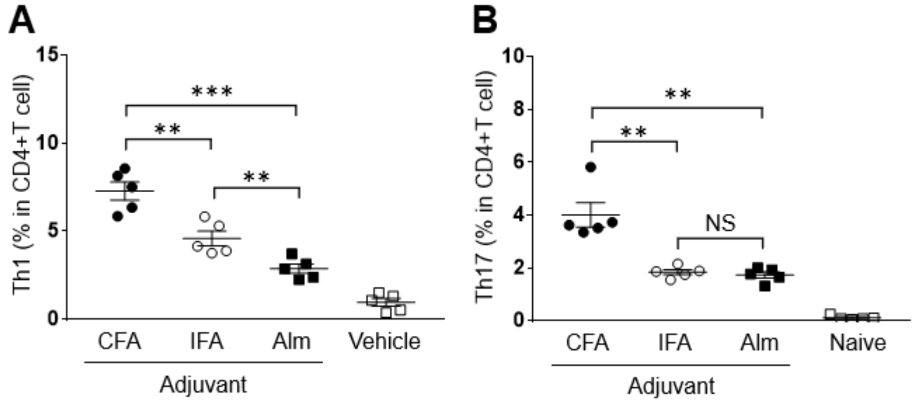

Figure S4. The difference of adjuvant effect in effector CD4+ T cell generation by SA-LP-F2 immunization. A-B) The mice were received SA-LP-F2 (10 ug in 50% of adjuvant) immunization into hind food pad. In the immunization, CFA, IFA or Aluminum (Alm) were used for adjuvant respectively in the each group. The mice received vehicle (PBS) injection were used for naive control. After 14 days, the mice received SA-LP-F2 (1 ug in 50% of adjuvant) IP injection. After 7 days (day 21 from initial immunizaion), the populations of Th1 (A) and Th17 (B) in spleen were analyzed by flow cytometry. Data are shown as the mean ± SEM of five samples. Two-way analysis of variance (ANOVA) was used to analyze data for significant differences. Values of **p<0.01, and ***p<0.001 were regarded as significant. NS; Not significant.

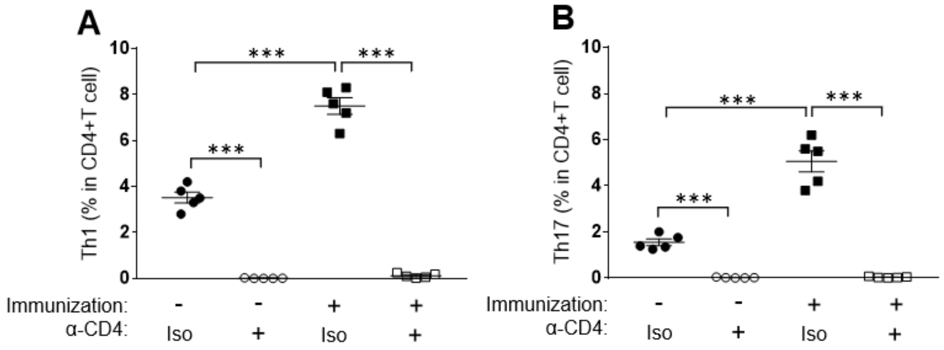

Figure S5. The efficiency of in vivo CD4+T cell depletion in SA-LP-F2 immunized mice. A-B) The mice were pre-treated with anti-CD4 mAb (500 ng) by IP injection from 3 days prior of SA-LP-F2 (10 ug) immunization (Immunization (+)). Isotype Ab was used for control mice (Iso). Some mice were received PBS injection as a vehicle control (Immunization (-)). After the immunization, the mice were consecutively received the anti-CD4 mAb (or isotype Ab for control) injection for every 3 days during experiment. After 14 days of the imminization, the mice were received SA-LP-F2 (1 ug/mL) IP injection. After 7 days (day 21 from initial immunizaion), the population of Th1 and Th17 in spleen were analyzed by flow cytometry. Data are shown as the mean ± SEM of five samples (A-B). Two-way analysis of variance (ANOVA) was used to analyze data for significant differences. Values of *p<0.05 , **p<0.01, and ***p<0.001 were regarded as significant.
